# Synthesis, structural determination and antimicrobial evaluation of two novel Co^II^ and Zn^II^ halogenometallates as efficient catalysts for the acetalization reaction of aldehydes

**DOI:** 10.1186/s13065-018-0393-6

**Published:** 2018-03-01

**Authors:** Assila Maatar Ben Salah, Lilia Belghith Fendri, Thierry Bataille, Raquel P. Herrera, Houcine Naïli

**Affiliations:** 1 Laboratoire Physicochimie de l’Etat Solide, Département de Chimie, Faculté des Sciences de Sfax, Université de Sfax, BP 1171, 3000 Sfax, Tunisia; 2 Unité Enzymes et Bioconversion, Ecole Nationale d’Ingénieurs de Sfax, PB 1173, 3038 Sfax, Tunisia; 3 Ecole Nationale Supérieure de Chimie de Rennes, 11 Allée de Beaulieu, 35708 Rennes cedex 7, France; 4 Laboratorio de Organocatálisis Asimétrica, Departamento de Química Orgánica, Instituto de Síntesis Química y Catálisis Homogénea (ISQCH), CSIC-Universidad de Zaragoza, C/Pedro Cerbuna 12, 50009 Saragossa, Spain

**Keywords:** Halogenometallate, X-ray diffraction, Thermal analysis, Antibacterial activities, Hydrogen bonds, Supramolecular architecture, Catalysis

## Abstract

**Background:**

Complexes of imidazole derivatives with transition metal ions have attracted much attention because of their biological and pharmacological activities, such as antimicrobial, antifungal, antiallergic, antitumoural and antimetastatic properties. In addition, imidazoles occupy an important place owing to their meaningful catalytic activity in several processes, such as in hydroamination, hydrosilylation, Heck reaction and Henry reaction. In this work, we describe the crystallization of two halogenometallate based on 2-methylimidazole. Their IR, thermal analysis, catalytic properties and antibacterial activities have also been investigated.

**Results:**

Two new isostructural organic-inorganic hybrid materials, based on 2-methyl-1*H*-imidazole, **1** and **2**, were synthesized and fully structurally characterized. The analysis of their crystal packing reveals non-covalent interactions, including C/N–H···Cl hydrogen bonds and π···π stacking interactions, to be the main factor governing the supramolecular assembly of the crystalline complexes. The thermal decomposition of the complexes is a mono-stage process, confirmed by the three-dimensional representation of the powder diffraction patterns (TDXD). The catalytic structure exhibited promising activity using MeOH as solvent and as the unique source of acetalization. Moreover, the antimicrobial results suggested that metal-complexes exhibit significant antimicrobial activity.

**Conclusion:**

This study highlights again the structural and the biological diversities within the field of inorganic–organic hybrids.

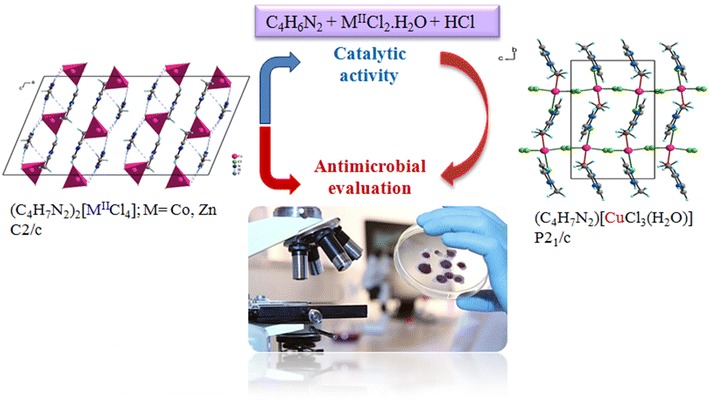

## Introduction

The chemistry of organic–inorganic hybrid materials constitutes one of the most flourishing areas of research in solid-state chemistry [1–3]. These hybrids are of interest because of their wide range of technologically advantageous properties, astounding compositional breadth, and exceptional diversity of structure. Thus, as a result of structural integration of organic cations and inorganic counterparts, magnetic [4–6], optical [7, 8], metallic conductivity [9] and catalytic [10, 11] properties have arisen in this class of chemical hybrid systems. Moreover, these materials may be used as model compounds for biological applications [12].

In our research, we particularly focus our attention on the preparation and the development of reactive transition metal complexes containing imidazole function for new, more selective or more widely catalytic and biological applications. Various metal complexes, especially these containing imidazole groups, occupy an important place owing to their meaningful catalytic activity in several processes, such as in hydroamination [13–16], hydrosilylation [17, 18] Heck reaction [19–23] and Henry reaction [24]. In addition, imidazoles play an important role in medicinal chemistry, because many of its derivatives have demonstrated significant biological activity. For example, in many metalloenzymes the imidazole rings of histidines play a pivotal role in metal-enzyme coordination. In consequence, the metal complexes of imidazoles have been widely used as model compounds of metalloenzymes [25–29]. It is well known that metal ions present in complexes accelerate the drug action and the efficacy of the organic therapeutic agents [30]. The pharmacological efficiencies of metal complexes depend on the nature of the metal ions and the ligands [31]. It is declared in the literature that different ligands and different complexes synthesized from same ligands with different metal ions possess different biological properties [30, 32, 33]. So, there is an increasing requirement for the discovery of new hybrid compounds having antimicrobial activities. However, this work has been quite selective. In this study, as an extension of our efforts into the development of new metal based antimicrobial complexes with 2-methylimidazole [34], we describe the crystallization of bis(2-methyl-1*H*-imidazolium)tetrachlorocobaltate(II) (C_4_H_7_N_2_)_2_[CoCl_4_] (**1**) and bis(2-methyl-1*H*-imidazolium)tetrachlorozincate(II) (C_4_H_7_N_2_)_2_[ZnCl_4_] (**2**), along with their crystal packing and crystal supramolecularity analyses. Their IR, thermal analysis, catalytic properties and antibacterial activities have also been investigated.

## Experimental section

### Materials

All the employed chemicals [Cobalt(II) chloride hexahydrate (CoCl_2_·6H_2_O), Zinc(II) chloride (ZnCl_2_), Hydrochloric acid (HCl; 37%) and 2-methyl-1*H*-imidazole (C_4_H_6_N_2_)] were commercial products (Sigma-Aldrich), which were used without further purification. All culture media and standard antibiotic were purchased from Bio-Rad laboratories, France).

### Synthesis

The two new compounds (C_4_H_7_N_2_)_2_[CoCl_4_] (**1**) and (C_4_H_7_N_2_)_2_[ZnCl_4_] (**2**) were obtained by slow evaporation, at room temperature. 2-Methyl-1*H*-imidazole (2mim) was dissolved with either CoCl_2_·6H_2_O or ZnCl_2_ in 10 mL of distilled water and hydrochloric acid HCl (pH ≈ 2.5) with the metal/amine molar ratio of 1:2. The clear solutions were stirred for 10 min until the complete dissolution and allowed to stand at room temperature. Transparent block crystals with the specific color of the metal appeared after few days. Then, the products were filtered off and washed with a small amount of distilled water before being dried in ambient air. Otherwise, they are also stable for a long-time in normal conditions of temperature and humidity.

### Single-crystal data collection and structure determination

Small crystals of the two compounds **1** and **2** were glued to a glass fiber mounted on a four-circle Nonius KappaCCD area-detector diffractometer with graphite monochromatized Mo Kα radiation, using an Oxford Cryosystems cooler. Data collection, absorption corrections frame scaling and unit cell parameters refinements were carried out with CrysAlisCCD and CrysAlisRED [35]. The structures analyses were carried out with the monoclinic symmetry, space groups C2/c, according to the automated search for space group available in Wingx [36]. Structures of **1** and **2** were solved with direct methods using SHELXS-97 [37] and refined by a full-matrix least squares technique with SHELXL-97 [37] with anisotropic thermal parameters for all non H-atoms. H atoms bonded to C and N atoms were positioned geometrically and allowed to ride on their parent atoms, with C–H = 0.95 Å and N–H = 0.88 Å. The drawings were made with DIAMOND program [38]. The main crystallographic data and refinement parameters are presented in Table 1.

**Table 1:** Crystal data and structure refinement details for (C_4_H_7_N_2_)_2_[CoCl_4_] (**1**) and (C_4_H_7_N_2_)_2_[ZnCl_4_] (**2**)

Compound	(**1**)	(**2**)
Chemical formula	(C_4_H_7_N_2_)_2_[CoCl_4_]	(C_4_H_7_N_2_)_2_[ZnCl_4_]
Compound weight	366.96	373.40
Temperature (K)	100 (2)	100 (10)
Crystal system	Monoclinic	Monoclinic
Space group	C2/c	C2/c
a (Å)	26.9330 (17)	26.871 (8)
b (Å)	7.8842 (2)	7.9031 (18)
c (Å)	15.0925 (5)	15.077 (5)
β (°)	111.001 (5)	111.23 (5)
V (Å^3^)	2991.9 (2)	2984.5 (15)
Z	8	8
ρ_cal_ (g cm^−3^)	1.629	1.662
Crystal dimension, mm^3^	0.45 × 0.37 × 0.13	0.50 × 0.42 × 0.12
Habit-colour	Block, blue	Block, transparent
μ (mm^−1^)	1.85	2.35
θ range (deg)	θ_min_ = 2.7, θ_max_ = 30.7	θ_min_ = 2.7, θ_max_ = 30.7
Index ranges	− 26 ≤ h ≤ 38	− 38 ≤ h ≤ 29
	− 10 ≤ k ≤ 10	− 9 ≤ k ≤ 11
	− 21 ≤ l ≤ 21	− 21 ≤ l ≤ 21
Unique data	4253	4197
Observed data [I > 2σ(I)]	3254	3731
F(000)	1480	1504
R_1_	0.053	0.050
wR_2_	0.132	0.121
GooF	1.012	1.17
No. param	156	157
Transmission factors	T_min_ = 0.334; T_max_ = 0.804	T_min_ = 0.387; T_max_ = 0.766
Largest difference map hole	Δρmin = − 1.11, Δρmax = 1.44	Δρmin = − 0.63, Δρmax = 2.36

### Infrared spectroscopy

All IR measurements were performed using a Perkin Elmer 1600FT spectrometer. Samples were dispersed with spectroscopic KBr and pressed into a pellet. Scans were run over the range 400–4000 cm^−1^.

### Thermal analyses

TGA–DTA measurements of **1** and **2** were performed on raw powders with a TGA/DTA ‘SETSYS Evolution’ (Pt crucibles, Al_2_O_3_ as a reference) under air flow (100 mL/min). The thermograms were collected on 9 mg samples in the temperature range from 25 to 650 °C (heating rate of 5 °C/min).

### Powder X-ray diffraction

The variable-temperature X-ray powder diffraction (VT-XRPD) for **1** and **2** was performed with a PANalytical Empyreanpowder diffractometer using CuKα radiation (λ*K*α_1_ = 1.5406 Å, λ*K*α_2_ = 1.5444 Å) selected with the Bragg–Brentano HD^®^ device (flat multilayer X-ray mirror) from PANalytical and equipped with an Anton Paar HTK1200N high-temperature oven camera. Powder X-ray diffraction was used to support the structure determination and to identify the crystalline phases of **1** and **2**. The thermal decompositions were carried out in flowing air from 20 to 670 °C. Patterns were collected every 7 °C, with a heating rate of 7 °C h^−1^ between steps.

### Catalytic studies

Complex **1** (4.7 mg, 0.01292 mmol) or **2** (4.8 mg, 0.01292 mmol) and aldehydes **3a**–**i** (0.323 mmol) were dissolved in MeOH (0.25 mL) in a test tube. The resulting mixture was stirred at 40 °C during 24 h. The reactions were monitored by thin-layer chromatography. The yield of the reaction is given by ^1^H NMR.

### Antimicrobial activity

Antimicrobial activity was essayed against three species of Gram negative bacteria [*Salmonella typhimurium* (ATCC 19430), *Pseudomonas aeruginosa* (ATCC 27853), *Klebsiella pneumonia* (ATCC 13883) and five species of Gram positive bacteria (*Enterococus faecalis* (ATCC 9763), *Bacillus thuringiensis* (ATCC 10792), *Staphylococcus aureus* (ATCC 25923), *Micrococcus luteus* (ATCC 4698) and *listeria*]. All microorganisms were stocked in appropriate conditions and regenerated twice before using.

Antimicrobial activity assays were performed according to the method described by Berghe and Vlietinck [39]. Steril enutrient agar medium was prepared and distributed into Petriplates of 90 mm diameter. A suspension of the previously prepared test microorganism (0.1 mL of 10^6^ UFCmL^−1^) was spread over the surface of agar plates (LB medium for bacteria). Then, bores (3 mm depth, 5 mm diameter) were made using a sterile borer and loaded with a concentration of 5 mg/mL of all samples. Before incubation, all petri dishes were kept in the refrigerator for 2 h to enable pre-diffusion of the substances into the agar. After that, they were incubated at 37 °C for 24 h. Ampicillin was used as positive reference. The diameters of the inhibition zones were measured using a ruler, with an accuracy of 0.5 mm. Each inhibition zone diameter was measured three times (in two different plates) and the results were expressed as an average of the radius of the inhibition zone in mm.

## Results and discussion

### Infrared spectra

**Fig. 1:**
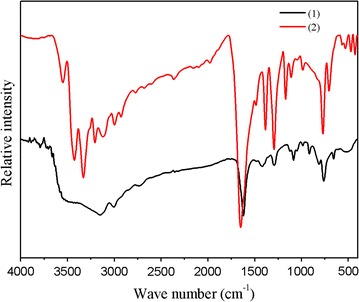
The infrared absorption spectra of compounds **1** and **2**, dispersed in a KBr pellet

The IR active bands of the 2-mim ring as well as the stretching vibrations of the N–H bond could be identified in the IR spectra of both compounds (Fig. 1). Indeed, it is known that the narrow bands at 3147.3 and 3120.9 cm^−1^, for **1** and **2** respectively, correspond to the ν_C–H_ stretching modes of the 2-mim ring [40]. Moreover the stretching vibration ν(NH) has been identified at 2724 and 3752 cm^−1^, for **1** and **2**, respectively. This agrees well with the structural study which proved the protonation of the 2-mim cation. The bands located in the region 1400–1650 cm^−1^ are assignable to C–C and C–N stretching vibration of 2-mim ring. The ν_C=N_ mode can be found at 1438 and 1492 cm^−1^ for **1** and **2**, respectively. Additionally, the vibrational bands from 1002 to 1438 cm^−1^ can be assigned to the ring stretching frequency of the 2-mim cation (ν_ring_) [41]. Finally, the bands remaining in the 686–859 cm^−1^ region can be associated with the deformations of the imidazole ring.

### Crystal structure

**Fig. 2:**
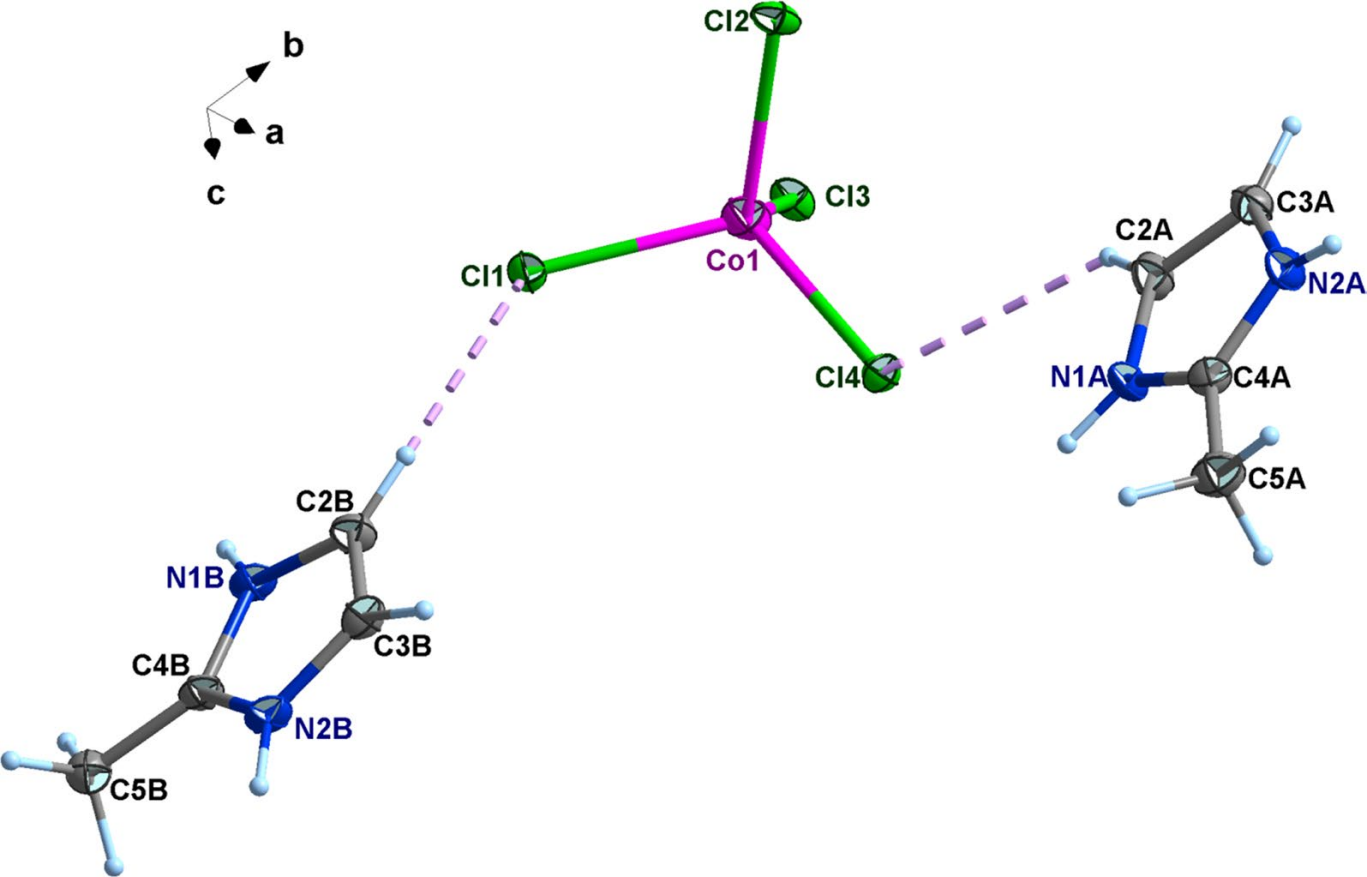
A view of the asymmetric unit cell of **1**. Displacement ellipsoids for non–H atoms are presented at the 50% probability level

Compounds **1** and **2** are isostructural, confirmed by their single crystal structural analyses (Table 1). Compound **1** was taken as an example to understand the structural details. Complex **1** crystallizes in the monoclinic centrosymmetric space group C2/c and its basic structure unit consists of one [CoCl_4_]^2−^ ion and two crystallographically inequivalent 2-mim cations, as shown in Fig. 2.

**Fig. 3:**
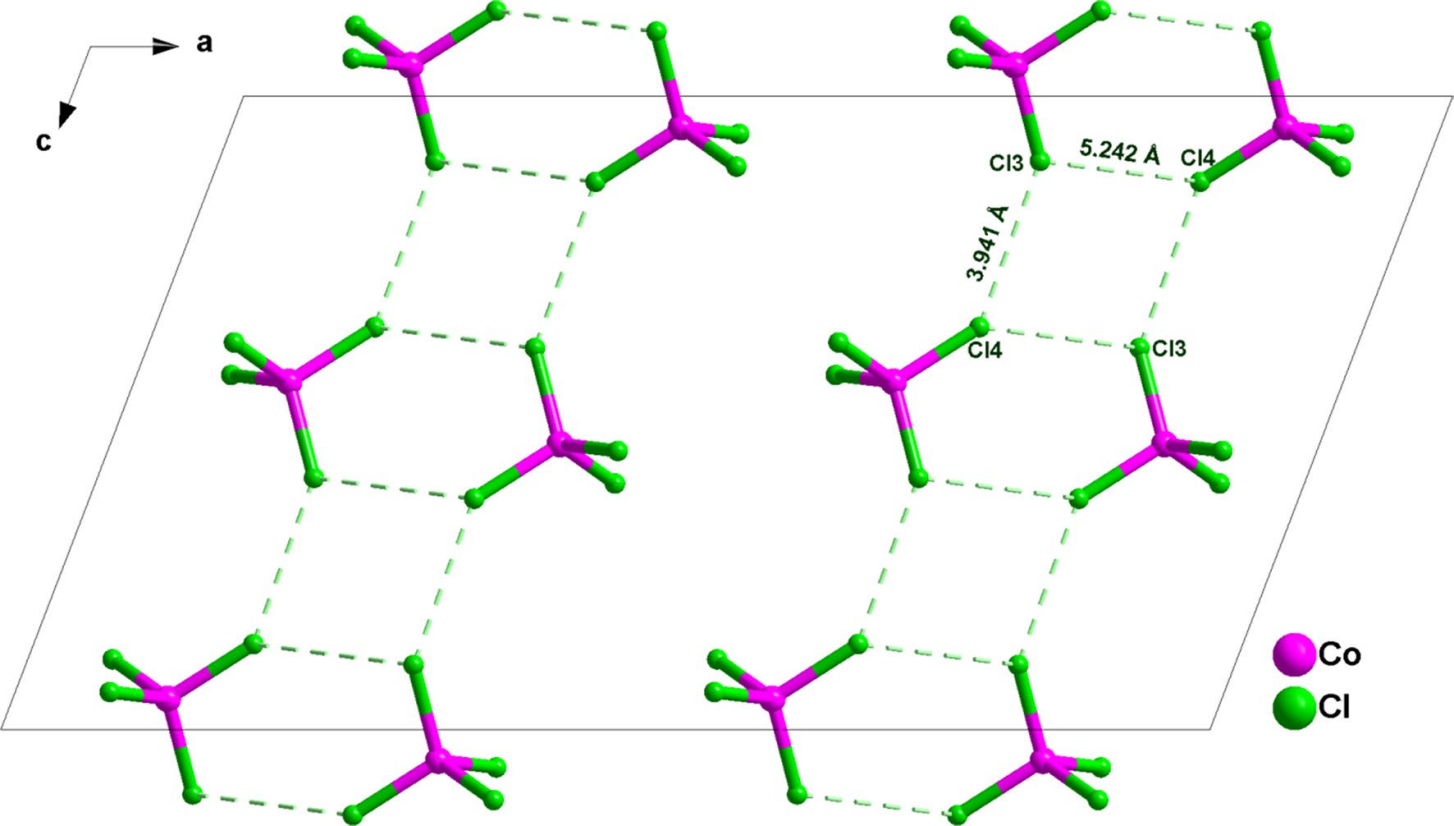
The Cl⋯Cl interactions within the mineral layers, showing its supramolecular aspect

The Co(II) ion is tetrahedrally bound by four chlorine atoms, with Co–Cl bond distances ranging from 2.246(9) to 2.287(9) Å and Cl–Co–Cl bond angles between 106.45(4)° and 111.87(3)°, which are slightly deviated from the ideal value of 109.28° (Table 2). Therefore, the coordination geometry around the Co^II^ ion can be described as a slightly irregular tetrahedron. Cobalt atoms are stacked one over the other along the three crystallographic axes and are isolated from each other with a shortest distance Co⋯Co = 7.330(4) Å which is more than the sum of the van der Waals radii of the cobalt ions tetrahedrally coordinated (4 Å). Hence, there is no metallophilic Co⋯Co interaction in this compound as proposed by Das et al. [42]. One of the main cohesive forces responsible for molecular arrangements of halogen derivatives is the pattern of halogen⋯halogen intermolecular interactions. It is worth mentioning here, that in bis(2-methyl-1*H*-imidazolium)tetrachlorocobaltate(II) the shortest Cl⋯Cl contacts between copper sites related by unit cell translations along the a or c directions are 5.242 and 3.941 Å, respectively, thus proving the weak halogen interactions in these directions (Fig. 3).

**Table 2:** Selected bond distances (Å) and angles (°) for **1** and **2**

Within the mineral moiety		Within the organic moiety	
(C_4_H_7_N_2_)_2_[CoCl_4_] (**1**)			
Co1–Cl1	2.2741 (9)	N1A–C4A	1.325 (5)
Co1–Cl2	2.2767(10)	N1A–C2A	1.380 (5)
Co1–Cl3	2.2870 (9)	N2A–C4A	1.327 (5)
Co1–Cl4	2.2464 (9)	N2A–C3A	1.372 (5)
Cl2–Co1–Cl1	106.45 (4)	N1B–C4B	1.331 (4)
Cl3–Co1–Cl1	108.69 (3)	N1B–C2B	1.374 (5)
Cl3–Co1–Cl2	110.55 (3)	N2B–C4B	1.331(4)
Cl3–Co1–Cl4	109.09 (4)	N2B–C3B	1.370 (5)
Cl2–Co1–Cl4	110.16 (4)	C2A–C3A	1.345(5)
Cl1–Co1–Cl4	111.87 (3)	C4A–C5A	1.479 (6)
		C2B–C3B	1.348 (5)
		C4B–C5B	1.478 (5)
		C4A–N1A–C2A	110.0 (3)
		C4A–N2A–C3A	110.1 (3)
		C4B–N2B–C3B	110.2 (3)
		C4A–N2A–C3A	110.1 (3)
		C3B–C2B–N1B	106.5 (3)
		C3A–C2A–N1A	106.3 (3)
		C2B–C3B–N2B	106.7 (3)
		C2A–C3A–N2A	106.8 (3)
		N1A–C4A–N2A	106.8 (3)
		N1A–C4A–C5A	126.5 (4)
		N2A–C4A–C5A	126.7 (4)
		N1B–C4B–N2B	106.5 (3)
		N1B–C4B–C5B	126.8 (3)
		N2B–C4B–C5B	126.6 (3)
(C_4_H_7_N_2_)_2_[ZnCl_4_] (**2**)			
Zn–Cl1	2.2780 (11)	N1A–C2A	1.325 (5)
Zn–Cl2	2.2779 (13)	N1A–C3A	1.381 (6)
Zn–Cl3	2.2392 (16)	N2A–C2A	1.335 (5)
Zn–Cl4	2.2945 (13)	N2A–C4A	1.386 (6)
Cl2–Zn–Cl1	106.45 (5)	C1A–C2A	1.438 (6)
Cl3–Zn–Cl1	112.11 (4)	C3A–C4A	1.347 (6)
Cl3–Zn–Cl2	110.49 (5)	N1B–C2B	1.336 (5)
Cl3–Zn–Cl4	109.43 (5)	N1B–C3B	1.372 (6)
Cl2–Zn–Cl4	109.98 (4)	N2B–C2B	1.332 (5)
Cl1–Zn–Cl4	108.31 (4)	N2B–C4B	1.380 (6)
		C1B–C2B	1.471 (6)
		C3B–C4B	1.348 (6)
		C3A–C4A–N2A	106.7 (4)
		C2A–N2A–C4A	109.8 (3)
		N1A–C2A–N2A	106.8 (4)
		N1A–C2A–C1A	126.6 (4)
		N2A–C2A–C1A	126.6 (4)
		C4A–C3A–N1A	106.7 (4)
		C2B–N1B–C3B	110.3 (4)
		C2B–N2B–C4B	110.3 (3)
		N2B–C2B–N1B	106.2 (4)
		N2B–C2B–C1B	126.6 (4)
		N1B–C2B–C1B	127.2 (4)
		C4B–C3B–N1B	106.8 (4)
		C3B–C4B–N2B	106.4 (4)

As far as the cation is concerned, all the bond lengths and bond angles observed in aromatic rings of the 2-mim present no unusual features and are consistent with those observed in other homologous derivates (Table 2) [40, 43]. The 2-methylimidazolium cation is essentially planar (maximum deviation from the mean plane through the imidazole ring is 0.0150 Å).

**Fig. 4:**
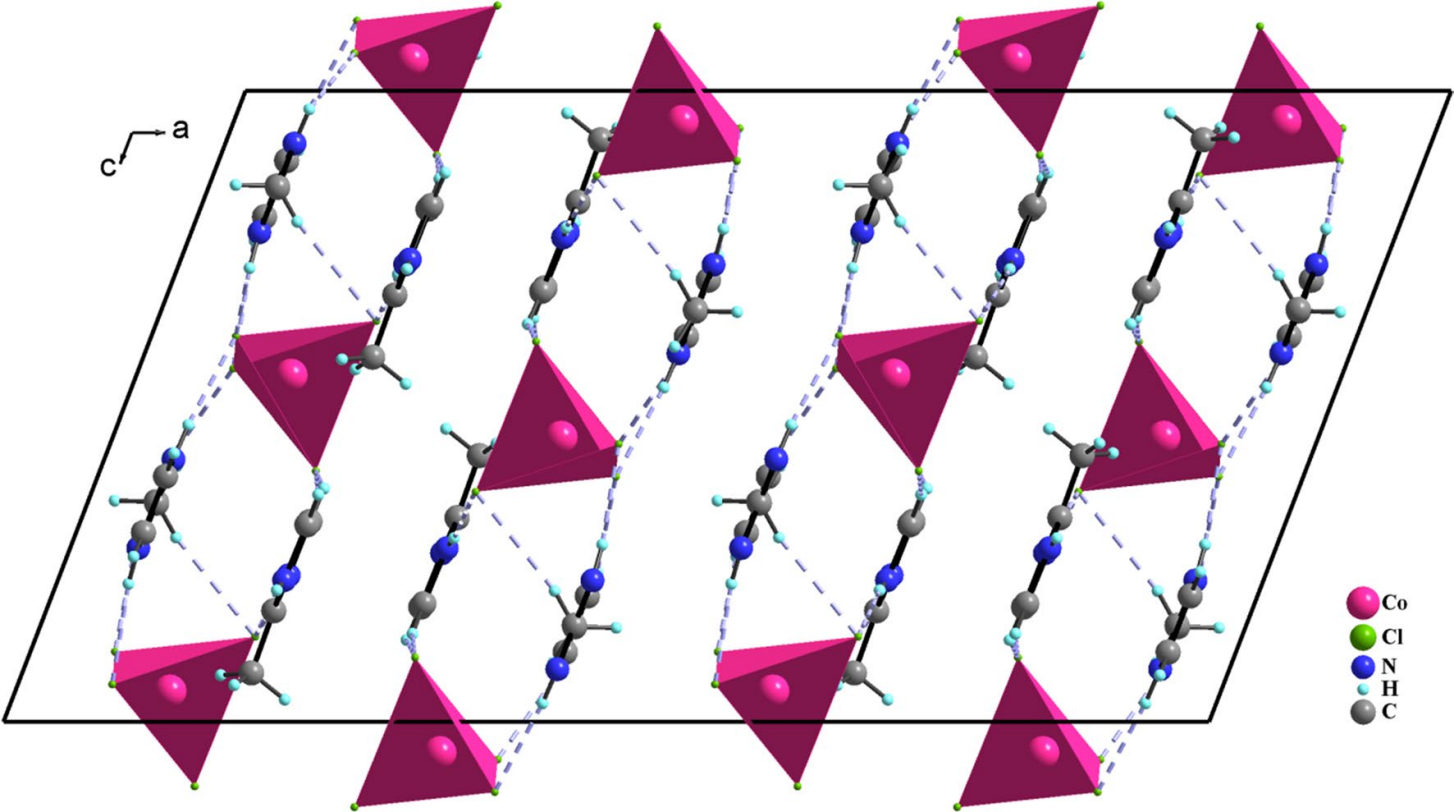
Projection of the structure of **1** along the crystallographic b axis, showing C/N–H⋯Cl hydrogen bonding between the inorganic and organic moieties

**Fig. 5:**
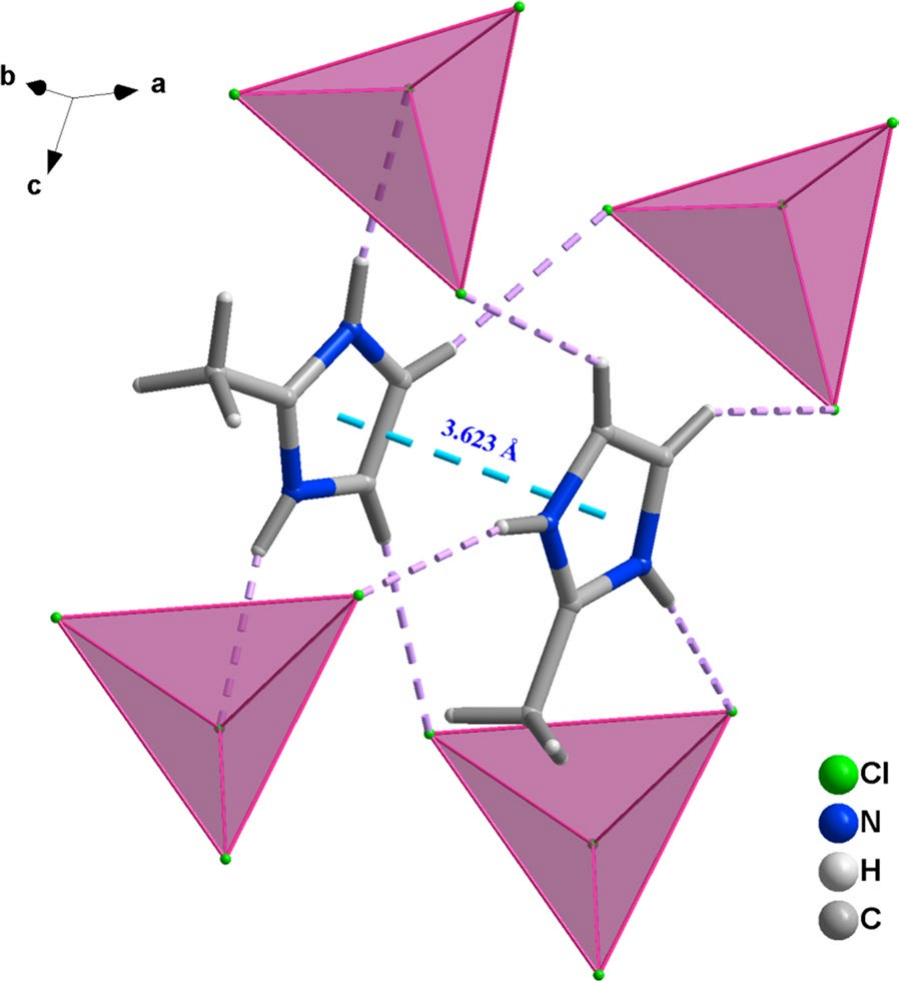
Crystal packing arrangement showing the π⋯π stacking interactions between the aromatic rings

The packing of the structure can be regarded as alternating stacks of anions and layers of cations. The isolated molecules are involved in many intermolecular interactions leading to layers that are parallel to bc plane (Fig. 4). These layers are stabilized and governed significantly through extensive C/N–H⋯Cl hydrogen bonding between the inorganic and organic moieties and π⋯π stacking interactions between the aromatic rings of the amine molecules themselves (Table 3). Indeed, the C⋯Cl distances vary from 3.422 (4) to 3.628 (4) Å, while the N⋯Cl distances vary from 3.160 (3) to 3.273 (3) Å. The centroid–centroid distance and dihedral angle between the aromatic rings are 3.62 Å and 0.00°, respectively, displaying typical π⋯π stacking interactions (Fig. 5). These values are almost comparable to the corresponding values for intermolecular π⋯π interactions, showing that π⋯π contacts may further stabilize the structure. Then, both C/N–H⋯Cl and π⋯π stacking interactions are the driving forces in generating a three-dimensional supramolecular network.

**Table 3:** Hydrogen-bonding geometry (Å, °) for **1** and **2**

D–H⋯A	d (D–H) (Å)	d (H⋯A) (Å)	d (D⋯A) (Å)	∠ D–H⋯A (°)
(C_4_H_7_N_2_)_2_[CoCl_4_] (**1**)				
N1A–H1A⋯Cl4	0.88	2.43	3.273 (3)	161
N2A–H2A⋯Cl4^i^	0.88	2.51	3.213 (3)	174
N1B–H1B⋯Cl1	0.88	2.31	3.188 (3)	173
N2B–H2B⋯Cl2^ii^	0.88	2.30	3.160 (3)	167
C2A–H2A1⋯Cl4	0.95	2.75	3.422 (4)	128
C3A–H3A⋯Cl4^i^	0.95	2.75	3.532 (4)	141
C2B–H2B1⋯Cl1	0.95	2.69	3.576 (4)	155
C3B–H3B⋯Cl2^ii^	0.95	2.71	3.628 (4)	163
(C_4_H_7_N_2_)_2_[ZnCl_4_] (**2**)				
N1A–H1A⋯Cl4^i^	0.88	2.34	3.222 (4)	176
N2A–H2A⋯Cl4	0.88	2.45	3.281 (4)	158
N1B–H1B⋯Cl1	0.88	2.30	3.158 (4)	164
N2B–H2B⋯Cl3^ii^	0.88	2.32	3.199 (4)	173

Symmetry codes for **1**: [(i) x, y − 1, z; (ii) x, − y, z − 1/2]; Symmetry codes for **2**: [(i) x, y + 1, z; (ii) x, − y − 1, z + 1/2]

### Thermal decomposition

**Fig. 6:**
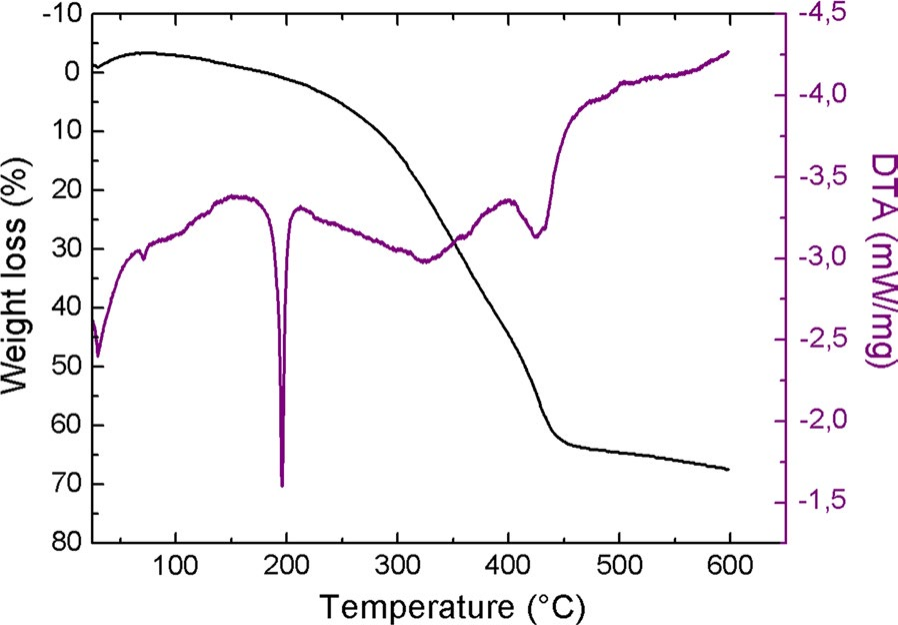
Simultaneous TG–DTA curves for the decomposition of **1**, under flowing nitrogen (5 °C/min from 25 to 650 °C)

**Fig. 7:**
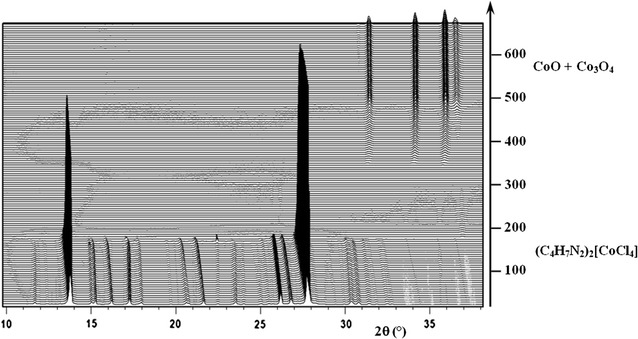
TDXD plot for the decomposition of **1** in air (7 °C h^−1^ from 20 to 670 °C)

The two compounds show similar thermal behavior, which further support their isomorphic structures. Thus, for simplicity the thermal properties of **1** only have been discussed. Thermogravimetric analyses of compound **1** were undertaken in the temperature range from 25 to 650 °C under flowing N_2_ atmosphere with a heating rate of 5 °C/min, leading to the simultaneous TGA/DTA profiles. The simultaneous (TG–DTA) curves and the three-dimensional representation of the powder diffraction patterns are shown in Figs. 6 and 7, respectively. As shown in Fig. 6, the small mass gain observed at room temperature on the TG curve is explained by the strong hygroscopic character of the sample, as also observed when the sample is ground for XRPD analysis. According to the TG curve, it is evident that compound **1** undergoes a single stage weight loss observed between 150 and 460 °C, accompanied by an intense endothermic peak at 195 °C and a shoulder endothermic peak at 425 °C, on the DTA thermogram. This mass loss corresponds to the elimination of the organic moiety and two chloride atoms, (observed weight loss, 64.01%, theoretical, 64.57%). This decomposition process is confirmed by the three-dimensional representation of the powder diffraction patterns (Fig. 7). Indeed, the TDXD plot reveals that the precursor, (C_4_H_7_N_2_)_2_[CoCl_4_], remains crystalline until 170 °C, while being subject to thermal expansion from room temperature, and then undergoes a complete structural destruction to become amorphous. The corresponding oxides, CoO and Co_3_O_4_, crystallize from 350 °C (ZnO for compound **2**).

### Catalytic study

The transformation of a carbonyl group into an acetal is one of the most recurrent methods for protecting carbonyl groups in organic synthesis [44]. However, although this is an extensive explored approach, it still presents some inconveniences that should be overcome [45–56]. Therefore, the development of new catalytic structures to successfully perform this protection is of high interest for the progress of this field. Despite the number of reported works regarding this reaction, to the best of our knowledge the use of Co- [57, 58] and Zn-based catalysts [59, 60] has been less explored in the literature until now. In this spectrum of properties, we envisioned the possibility of testing the effectiveness of our metallic species in the acetalization reaction of aldehydes as a benchmark process.

In order to explore the efficiency of both candidates, we firstly tested their activity in the model acetalization reaction depicted in Table 4. Both catalytic structures shown the same order of reactivity at room temperature (compare entries 1–4 and 6–9). With a more concentrated reaction medium and 4 mol% of catalyst better yields are obtained (compare entries 2 and 4, and entries 7 and 8). At 40 °C catalyst **2** exhibited a slightly better reactivity with an almost complete conversion of the process (compare entries 5 and 10). Although CH(OMe)_3_ is the commonly used source of acetalization in the protection of carbonyl compounds, interestingly, only MeOH is used in our protocol as the most accessible source.

**Table 4:** Screening of the reaction conditions to optimize the acetalization process

Entry	Complex (mol%)	MeOH (mL)	Temperature (°C)	Yield (%)^a^
1	**1** (2)	0.25	r.t.	65
2	**1** (4)	0.25	r.t.	78
3	**1** (6)	0.25	r.t.	78
4	**1** (4)	0.50	r.t.	59
5	**1** (4)	0.25	40	92
6	**2** (2)	0.25	r.t.	63
7	**2** (4)	0.25	r.t.	83
8	**2** (6)	0.25	r.t.	71
9	**2** (4)	0.50	r.t.	67
10	**2** (4)	0.25	40	97

Otherwise indicated: a mixture of aldehyde **3a** (0.323 mmol) and catalysts **1** or **2** (4 mol%) in 0.25 mL MeOH, was stirred at 40 °C for 24 h. After this time the reaction crudes were analysed by ^1^H NMR

^a^Yields of **4a** [61] determined by ^1^H-NMR spectroscopy

After this screening, and with the best reaction conditions in hand, we extended our strategy to different substituted aldehydes as shown in Table 5. As reported in Table 5, the desired acetals **4b**–**i** were obtained with very good yields. The developed methodology was successfully applied to all aromatic aldehydes examined **3a**–**i** giving rise to really clean reaction crudes. Interestingly, neither inert atmosphere nor dry or other special conditions were needed to carry out the reactions. As a proof of fact, the reactions were performed in the absence of catalysts, demonstrating the efficiency of our catalytic species, since no reaction was observed in the background processes (< 5%). It seems that the electronic effects over the aromatic ring affects to the reactivity of the process, since activated aldehydes, with electron-withdrawing groups in their structure, rendered better yields in comparison with non-activated ones (compare entries 1–5 with entries 6–8). Further catalytic studies are actually ongoing in our laboratory in order to explore additional reactions with both catalytic species.

**Table 5:** Scope of the acetalization reaction using catalyst **2**

Entry	R	Product	Yield (%)^a^
1	4-ClPh, **3b**	**4b** [62]	93
2	3-ClPh, **3c**	**4c** [62]	81
3	4-BrPh, **3d**	**4d** [62]	81
4	4-NO_2_Ph, **3e**	**4e** [62]	94
5	4-CNPh, **3f**	**4f** [63]	94
6	4-PhPh, **3** **g**	**4g** [64]	67
7	Ph, **3** **h**	**4** **h** [62]	75
8	1-Naphthyl, **3i**	**4i** [62]	70
9^b^	3-NO_2_Ph, **3a**	**4a**	< 5

^a^Yields determined by ^1^H-NMR spectroscopy

^b^Reaction performed in the absence of catalyst

**Scheme 1:**
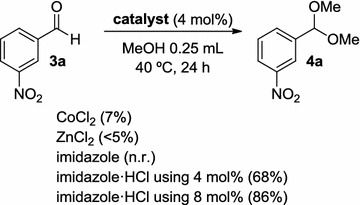
Control experiments (*n.r.* no reaction observed)

**Scheme 2:**
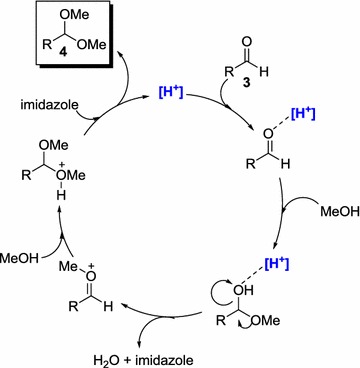
Tentative mechanistic cycle

In order to gain insight about the most active specie of our structures, we carried out some control experiments using the simplest species described in Scheme 1. In this sense, CoCl_2_ and ZnCl_2_ were used as direct precursors of the crystalline structures **1** and **2**, under the best reaction conditions above described in Table 5. Surprisingly, these metal species did not provide the acetalization reaction of aldehyde **3a**. Then, we focused on the contra ion of the crystal structures **1** and **2**, that is an imidazolium cation. First, imidazole was tested in the reaction as plausible catalyst but the reaction did not work as expected, since this process is acid promoted. In contrast, the generated chlorohydrate salt activated the reaction in a 68% as a weak acid catalyst. Since the crystal structures **1** and **2** bear two imidazolium molecules, the use of an 8 mol% of the acidic specie was also explored giving rise to an 86%. The activity observed with the imidazolium salt supports that the reactivity found with catalysts **1** and **2** is directly related with this salt species instead of the metal atom. This finding is also in agreement with the similar results observed when both species, **1** and **2**, were initially screened (Table 4). However, at this point we cannot discard a plausible synergic effect of the whole complex structure, since the results obtained with catalysts **1** and **2** are slightly better (Table 4, entries 5 and 10, respectively) than the results obtained just with imidazolium salt (Scheme 1). A possible acidification of the most acid proton in the imidazolium structure as a result of the interaction with the metal complex anion could be tentatively suggested. Although more studies should be necessary to support the mechanism of this process using complexes **1** and **2**, a plausible catalytic cycle is proposed in Scheme 2 and the role of imidazolium salt, represented as H^+^, is depicted. The weak acid would be involved in the first step of the cycle activating the aldehyde to promote the addition of the first molecule of MeOH [65].

### In-vitro antimicrobial activity

In this part and by way of comparison, we chose to study the antibacterial activity of the organic–inorganic hybrid metal(II) halides with 2-methylimidazole. Then the synthesized compounds as well as the copper complex based on 2-mim, recently published [34], were screened for their in vitro growth inhibiting activity against Gram-positive (*Enterococus faecalis*, *Bacillus thuringiensis*, *Staphylococcus aureus*, *Micrococcus luteus* and *listeria*) and Gram-negative (*Salmonella typhimurium*, *Pseudomonas aeruginosa* and *Klebsiella pneumonia*) bacteria. The antibacterial activity was measured as the diameter of the clear zone of growth inhibition and the results were presented in Table 6. As can be seen in this table, (C_4_H_7_N_2_)_2_[CoCl_4_] (**1**), (C_4_H_7_N_2_)_2_[ZnCl_4_] (**2**) and (C_4_H_7_N_2_)[CuCl_3_(H_2_O)] (**3**) possessed variable inhibition zones among the tested microorganisms ranging from 11 to 24 mm at the tested concentration (5 mg/mL). Cobalt complex was found to have a significant antibacterial activity against the Gram-negative bacteria tested compared to Gram-positive bacteria. In fact, compared to the ampicillin, (C_4_H_7_N_2_)_2_[CoCl_4_] has the same diameter inhibition zones (24 mm) against *K. pneumoniae*. (C_4_H_7_N_2_)_2_[CoCl_4_] exhibits a greater activity (20 mm) than the ampicillin against *S. Typhimurium*. According to the results presented in Table 6, (C_4_H_7_N_2_)_2_[ZnCl_4_] was found to have a moderate activity against *E. faecalis*, *P. aeruginosa* and *S. Typhimirium.* No inhibition zones were observed for all the tested chemical compounds against *B. thuringiensis* and *M.l*. In fact, from these results can be deduced that these two Gram-positive bacteria were found to be very resistant. The antimicrobial results suggested that Co-complex exhibits higher biologically activity against microbial tested strains in comparison to the ampicillin antibiotic.

**Table 6:** Antibacterial activity of **1**, **2** and **3** against Gram (+) and Gram (−) bacteria strains

Bacteria strains	Inhibition zone diameter (mm)			
	**1**	**2**	**3**	Ampicillin
Gram +				
*S. aureus*	11 ± 0.5	nd	nd	40 ± 0.5
*E. faecalis*	13 ± 0.5	13 ± 0.5	nd	26 ± 0.5
*Listeria*	15 ± 0.5	nd	13 ± 0.5	33 ± 0.5
*B. thuringiensis*	nd	nd	nd	36 ± 0.5
*M. l*	nd	nd	nd	20 ± 0.5
Gram −				
*K. pneumoniae*	24 ± 0.5	nd	11 ± 0.5	24 ± 0.5
*P. aeruginosa*	13 ± 0.5	11 ± 0.5	nd	23 ± 0.5
*S. Typhimirium*	20 ± 0.5	12 ± 0.5	nd	15 ± 0.5

*nd* not detected

## Conclusions

Two new isostructural organic–inorganic hybrid materials, based on 2-methyl-1*H*-imidazole, **1** and **2**, were synthesized and fully structurally characterized. The basic unit structure of these compounds consists of one [M^II^Cl_4_]^2−^ ion and two crystallographically inequivalent 2-methylimidazolium cations. Furthermore, the analysis of their crystal packing reveals non-covalent interactions, including C/N–H⋯Cl hydrogen bonds and π⋯π stacking interactions, to be the main factor governing the supramolecular assembly of the crystalline complexes. In view of the pivotal role of noncovalent interactions in the design of new materials, the theoretical calculation method of noncovalent interactions (NCI) is found to be an effective tool to understand the formation of these complex materials. The thermal decomposition of the complexes is a mono-stage process, confirmed by the three-dimensional representation of the powder diffraction patterns (TDXD). Additionally, we have demonstrated the efficiency of both metal complexes to act as catalysts in the acetalization reaction under very mild conditions in the presence of MeOH, as the solvent of the reaction and as the unique source of acetalization. Moreover, the antimicrobial results suggested that metal-complexes exhibit significant antimicrobial activity. This study, in conjunction with the previous one [34], highlights again the structural and the biological diversities within the field of inorganic–organic hybrids.

## Abbreviations

2mim: 2-methyl-1H-imidazole
DTA: differential thermal analysis
TGA: thermogravimetric analysis
IR: infrared
XRPD: X-ray powder diffraction
